# Zebrafish as an Experimental Model for Human Disease

**DOI:** 10.3390/ijms24108771

**Published:** 2023-05-15

**Authors:** Federica Tonon, Gabriele Grassi

**Affiliations:** 1 Department of Medical, Surgical and Health Sciences, University of Trieste, Cattinara Hospital, Strada di Fiume 447, I-34149 Trieste, Italy; effe.tonon@gmail.com; 2 Department of Life Sciences, Cattinara University Hospital, University of Trieste, Strada di Fiume 447, I-34149 Trieste, Italy

Belonging to the family of Cyprinidae, the zebrafish is a small freshwater fish present in the rivers of Bangladesh, Northern India and Southern Nepal [1]. Active during the day, in the wild, zebrafish feed on small aquatic invertebrates. Their use as a model organism to investigate novel therapeutic molecules as well as human pathologies was firstly proposed by Streisinger et al. in 1981 [2]. There are a number of reasons for its employment in the bio-medical research field [3,4]. Zebrafish can produce many eggs, which take a short time to develop: gastrulation begins 6 hours post-fertilization (hpf) and hatching occurs 2 days later with the generation of free-swimming larvae. Thus, it is possible to obtain large amounts of larvae/adult animals in a short amount of time. Despite the great number of animals generated, little space is needed for housing zebrafish as the embryos/adults are of a small size. Thus, compared to other bigger animal models, the cost of maintaining zebrafish is definitively more manageable. Another remarkable feature can be attributed to the transparency of the larvae body: this allows researchers to easily track the position of labeled cells injected in the body. To overcome the tendency of the adult body to lose transparency, a genetic strain has been developed which is able to help the fish to maintain much of this property [5]. Particularly useful for the generation of xenograft models of human diseases is the fact that zebrafish lack an adaptive immune response until about four weeks post-fertilization. This feature allows researchers to bypass the problem of the rejection of injected cells [6]. Moreover, compared to other animal models, a very small number of cells (about a few hundred cells) is required to generate the xenograft. Notably, xenograft generation is possible despite the fact that zebrafish larvae develop best at 28 °C, while human cells need a temperature of 37 °C. It has been observed that it is possible to maintain zebrafish at 35 °C, allowing the survival of both animal and human xenograft cells [7]. Finally, it should be pointed out that zebrafish share many molecular signaling pathways with humans. Together, the above features make zebrafish particularly suited to be employed as animal models in bio-medical research. Since 1981 [2], the zebrafish has been employed in many different biomedical studies. For example, it is possible to study the effects of light on DNA replication in zebrafish [8], to generate xenograft models of human hepatocellular carcinoma [9,10,11], to study the effects of bacterial toxin [12] and many other applications.

The remarkable contributions included in the present Special Issue demonstrate that the zebrafish animal model is suited to study a great variety of human pathological conditions. This Special Issue entitled “Zebrafish as an Experimental Model for Human Disease” of the *International Journal of Molecular Sciences* includes a total of eight contributions: four original articles and four reviews providing novel information about the employment of zebrafish in human tumor, cardiac, neuro and metabolic diseases.

Rojas et al. [13] explored the therapeutic potential of two Ruthenium(II) arene complexes named JHOR11 and JHOR10 in the ovarian carcinoma cell line A2780, colorectal carcinoma cell line HCT116, doxorubicin-resistant HCT116 (HCT116-Dox) and in normal human dermal fibroblasts, used as the control. In vitro, JHOR10/JHOR11 had a good anti-proliferative effect on A2780 and HCT116 cell lines (range of maximal effect in the different cell lines: 7.5–50 μM). Notably, JHOR11 also displayed a good anti-proliferative effect on HCT116-Dox. This observation may contribute to address the frequent occurrence of drug resistance in colorectal carcinoma cells. Notably, both compounds had no significant effects on the control human dermal fibroblasts, suggesting a minor impact on normal cells. Due to the higher internalization of JHOR11 compared to JHOR10, other aspects in JHOR11-treated cells were also evaluated, such as cell death, migration and oxidative stress activation. The results suggested that JHOR11 did not promote the intrinsic apoptotic pathway but rather an autophagic cell death mechanism in A2780 cells. Since the cytotoxicity of Ruthenium(II) complexes are commonly associated with oxidative stress, the reactive oxygen species (ROS) was also quantified in A2780-treated cells. The results showed a significant increase in intracellular ROS concentration in JHOR11-treated cells compared to those exposed to the vehicle. JHOR11 also showed cytostatic effects by inducing an increase in G2/M phase cells compared to those treated with the vehicle (DMSO). In contrast, no JHOR11-induced anti-migratory and anti-proliferative effects were observed. Also interesting was the observation that, up to 80 μM of drug, negligible effects on zebrafish survival were observed. This dose was used to study the effect of 300 HCT116 cells labeled with Dil lipophilic dye and injected into the vein system (duct of Cuvier) 48 hpf of the animals. JHOR11 was dissolved in the embryo water at 1 day post-injection (dpi, 72 hpf). The visualization of the cells in the animal tail was performed at 4 dpi (144 hpf), showing a decrease in cell proliferation by 45% compared to the control. This work represents an excellent example of the use of zebrafish as a testing model for drug effectiveness and safety.

Pancreatic ductal adenocarcinoma (PDAC) is a terrible disease with an overall 5-year survival rate of less than 8%. Wang et al. [14] explored the effectiveness of different drugs against PDAC cells as well as the interaction of tumor cells with the immune system. For this purpose, the authors used four different human PDAC cell lines (PANC1, BxPC3, AsPC1 and CFPAC1) to generate the zebrafish xenograft model. PDAC cell lines labeled with the Dil dye were injected independently into the yolk sac of 48 hpf embryos. At 4 dpi (144 hpf), all cell lines were efficiently engrafted, but the AsPC1 tumor grew significantly more than the PANC1 and BxPC3 tumors. For PANC1 and AsPC1, the ability to form agglomerates in the intestine and the caudal hematopoietic tissue was also noted, thus showing the metastatic capability. The administration of the anti-cancer drugs irinotecan or 5-Fluoro-Uracile (5-Fu) resulted in a remarkable reduction in cell growth for all tumor cell types (Irinotecan) or for BxPC3/AsPC1 only (5-Fu). To study the interaction of the cancer cells with the innate immune response of zebrafish, the authors generated zebrafish xenograft models using the following zebrafish strains: (I) Tg(coro1a: GFP), where the innate immune cells (neutrophils, macrophages and thymus lymphocytes [15]) express the green fluorescence protein (GFP), (II) Tg(mpeg1: GFP) and (III) Tg(mpx: GFP), where macrophages or neutrophils are labeled with GFP, respectively. Using this approach, it was possible to study the interaction of tumor cells (stained with the red Dil dye) and the different immune cells (expressing GFP). The authors demonstrated that the primary-tumor-derived cells PANC1/BxPC3 activated the innate immune anti-tumoral responses, while the metastatic cells AsPC1/CFPAC1 suppressed the innate immune system. Notably, PANC1 and BxPC3 recruited more innate immune cells (coro1a:GFP) to the tumor micro-environment compared to the AsPC1 cells, and recruitment increased over time. In contrast, PANC1, BxPC3 and AsPC1 recruited a comparable number of neutrophils (mpx:GFP+) to the tumor area, and recruitment did not change from 1 dpi to 4 dpi for all cell lines. Therefore, the authors suggested that macrophages might be the predominant populations in coro1a: GFP+ innate immune cells recruited in the tumor area. This hypothesis should be proven using reporter lines (i.e., mpeg1: mCherry-F) to better label the macrophages. However, in the zebrafish strain irf8−/−, which decreased macrophages but increased neutrophils, the tumor mass of PANC1 and BxPC3 cells slightly increased, whereas the AsPC1-xenografted cells showed a significant growth reduction, suggesting that macrophages may drive the innate immune response in the tumor micro-environment. This elegant work clearly shows how the zebrafish model can be profitably used to study complex relation(s) between tumor cells and the immune system.

The employment of the zebrafish model is not limited to tumor investigation studies; it can also be adopted as a useful research tool in the field of neuro-muscle diseases. In this regard, Voisard et al. [16] studied the biological role of Valosin-containing protein (VCP), an ATPase enzyme involved in the regulation of protein quality control, turnover and degradation [17]. Defects (mutations) of VCP can cause different cardio-myopathy and neurodegenerative diseases in humans. The authors generated a constitutive VCP knockout zebrafish model by resorting to the use of the CRISPR/Cas9 system. This novel model is of particular significance as, due to the embryonic lethality, no other constitutive VCP knockout animal models are available. The zebrafish animal knockout for VCP had reduced cardiac and skeletal muscle function due to the disruption of myofibrillar organization and the accumulation of inclusion bodies. Moreover, mitochondrial degeneration was observed. All of these observations may be dependent on the accumulation of ubiquitinated proteins, not properly eliminated by the proteasome complex. The zebrafish model developed will be extremely important in studying the mechanistic underpinnings of VCP loss.

Diogo et al. [18] employed zebrafish to study a particular aspect of diabetes related to male impaired reproduction ability. Diabetes mellitus, a metabolic disorder characterized by chronic hyperglycemia, is classified as type I or type II depending on a deficient production of the hormone insulin or acquired insulin resistance, respectively. While type I typically affects young patients, type II belongs to adult/old patients. Diabetes is a very common pathology expected to affect more than 500 million peoples by 2035. Diabetes is responsible for accelerated micro- and macroangiopathy which can bring about multiple organ failure and death. Additionally, diabetes impairs the male reproductive system as glucose metabolism plays a relevant role in spermatogenesis and spermatozoa metabolism. In a zebrafish model of diabetes type I, the authors observed significantly lower sperm motility, plasma membrane viability and DNA integrity compared to those of a control group. This was paralleled by the significantly increased expression of insulin and glucose transporter compared to the controls. Whilst these observations need further investigation and a mechanistic connection with the spermatozoa phenotype observed, they indicate the suitability of zebrafish for studying the effects of type I diabetes mellitus on the male reproductive function.

Dougnon et al. [19] illustrate in their review the possibility of using zebrafish as a model to study two neurodevelopmental disorders, i.e., autism spectrum disorder (ASD) and attention-deficit/hyperactivity disorder (ADHD). Despite being considered two distinct diseases, patients affected by ASD and ADHD have in common the difficulty of paying attention and problems related to concentration, activity and relationships. Whilst little information about the pathogenesis of both ASD and ADHD is available, it is generally assumed that genetics can play a relevant role. Thus, different zebrafish models bearing defined genetic defects have been generated and their behavior has been evaluated by means of specific tests. For example, it is possible to evaluate the attitude to stay with other fish (social preference tests), their shoaling propensity (shoaling propensity test), their impulsiveness and attention (five-choice serial reaction time task), their condition of hyperactivity and anxiety-like phenotypes (the novel tank test) and aggressiveness (the mirror-attack test). Thus, starting from a given genetic defect, it is possible to study the corresponding phenotypic behavior. Obviously, this approach has to be coupled with the characterization at the molecular level of the alteration induced by a given genetic defect. Thus, the review of Dougnon et al. underlines the suitability of zebrafish to study very complex diseases such as ASD and ADHD.

The appropriateness of using zebrafish to study nervous system diseases is supported by their strict anatomical and physiological similarity with mammals. Rosa et al. [20] in their review show that the major neurotransmitter systems such as glutamate, gamma-aminobutyric acid (GABA), acetylcholine, dopamine, serotonin and noradrenaline (NA) are similar between zebrafish and mammals. Like in mammals, glutamate regulates synaptic transmission and neuronal excitability. GABA-ergic neurons are present in different regions of the zebrafish nervous system, such as the olfactory bulb, cerebellum and medulla oblongata. Acetylcholine modulates cognitive processes. Dopamine controls the locomotion of zebrafish larvae and the manipulation of the dopaminergic system results in behavioral phenotypes similar to those observed in rats and humans. Serotonin regulates aggression, anxiety, cognition and sleep. NA regulates the autonomic nervous system and controls learning and memory. These observations represent the rationale for the use of zebrafish to reproduce complex behavioral models able to mirror those of human neurological disorders including Alzheimer’s disease, Parkinson’s disease, depression/anxiety and epilepsy.

The employment of a zebrafish model in the field of neuroscience is not limited to the above reported diseases; it extends to the effects that metabolic syndrome can have on brain homeostasis. This is the subject of the review of Ghaddar et al. [21], with the focus being placed on the effects of obesity and diabetes on brain homeostasis. Metabolic syndrome is a pathological condition characterized in humans by a combination of at least three of the following alterations: abdominal obesity, hypertriglyceridemia, low serum high-density lipoprotein (HDL) levels, hyperglycemia (associated with insulin resistance) and hypertension. The authors elegantly describe the different models of metabolic disorders available in zebrafish (models of acute and chronic hyperglycemia and models of being overweight and obesity leading to hyperglycemia). Subsequently, they discuss the use of these models to study the pathological effects on the brain of fish. The zebrafish is particularly suited for this investigation as this fish possesses the blood–brain barrier (BBB) that helps in maintaining brain integrity, like as in humans; additionally, the zebrafish nervous system has high regenerative capacity. The authors report that, for example, under a hyperglycemic condition, BBB damage occurs leading to the manifestation of brain oxidative stress and neuro-inflammation. This in turn can bring about neurodegeneration with the development of cognitive impairments (locomotion, anxiety, memory) and neurodegenerative diseases. The authors also underline the fact that it is possible to examine in the zebrafish models the molecular mechanisms leading to the pathological phenotype.

Russo et al. [22] in their review discuss the suitability of the zebrafish for investigating the effects of obesity on the whole body. It should be noted that the World Health Organization (WHO) defines obesity as one of the main health concerns of the 21st century. As observed for the nervous system, the zebrafish is similar to humans with regard to the digestive organs, adipose tissue, liver and skeletal muscle, all involved in obesity. Like in humans, the zebrafish has appetite regulation in the brain, can control insulin release and lipid storage and has similar metabolic pathways to regulate adipocyte differentiation, energy homeostasis and cholesterol metabolism. Moreover, the zebrafish responds well to diet modifications, has high triglyceride levels and hepatic steatosis determined by excessive nutrient intake and lipids are accumulated both in visceral, intramuscular and subcutaneous adipose tissue, thus allowing us to examine body fat distribution in its globality. However, some differences with humans exist, such as a lack of thermoregulation and brown adipose tissue. Despite this, zebrafish are used to study the effects of anti-obesity drugs such as flavonoids, as reported by Russo et al. in their review.

The experimental works and the reviews of this Special Issue, together with the previous evidence, strongly point toward the suitability of zebrafish in studying several human diseases. Apart from the technical advantages described in the beginning of this editorial, the suitability of this model relies on the anatomical/molecular similarity with humans. This is rather surprising considering that the common ancestor between fishes and mammals dates back to several hundreds of millions of years. We trust that in the future zebrafish will be increasingly considered as a valuable tool to dissect the molecular aspect of human diseases as well as to test drug effectiveness. However, since some evident differences exist with mammals, we believe that the zebrafish could be potentially considered as a first-line testing model whose employment could reduce the use of more complex and costly animal models.
